# Adsorption Studies of Dilute Krypton and Xenon from Nitrogen on SBMOF‑1 and Activated Charcoal for Applications in Isotope Harvesting

**DOI:** 10.1021/acs.jpcc.5c02812

**Published:** 2025-07-21

**Authors:** Vladyslav S. Bodnar, Chloe R. Kleinfeldt, Sung Ho Kim, Noelle R. Catarineu, Chirag K. Vyas, Ate Visser, Gregory W. Severin

**Affiliations:** † Department of Chemistry, 242484 Michigan State University, East Lansing, Michigan 48824, United States; ‡ Facility for Rare Isotope Beams, Michigan State University, East Lansing, Michigan 48824, United States; § 4578 Lawrence Livermore National Laboratory, Livermore, California 94550, United States

## Abstract

Adsorptive partitioning of dilute krypton (Kr) and xenon (Xe) onto Stony Brook Metal–Organic Framework (SBMOF-1) and activated charcoal (AC) from carrier nitrogen was experimentally measured at temperatures ranging from 195 to 293 K. For this purpose, a closed-loop system for gas adsorption experiments was developed. From the Kr adsorption measurements, the adsorption equilibrium constant for Kr on SBMOF-1 was calculated, yielding a value for the enthalpy of adsorption of −19 ± 1 kJ·mol^–1^. The partition coefficients were utilized to estimate the extraction rates of ^76^Kr, ^77^Kr, and ^122^Xe isotopes during isotope harvesting at the Facility for Rare Isotope Beams (FRIB). We conclude that both materials showed promising results for the extraction of noble gases from FRIB effluents using temperature swing adsorption.

## Graphical abstract

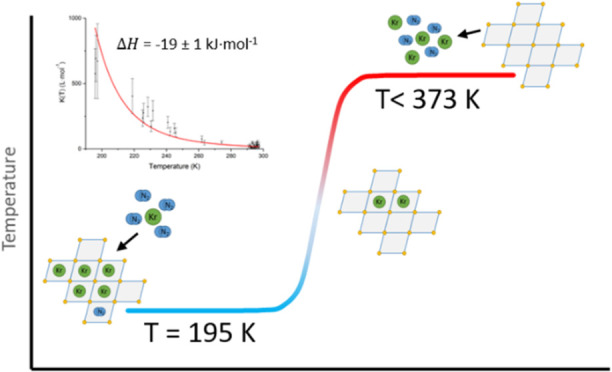

## Introduction

At the Facility for Rare Isotope Beams (FRIB), useful quantities of radioactive noble gases such as ^76^Kr, ^77^Kr, and ^122^Xe will be produced in a water-filled beam dump during routine operations.^1,2^ Developing a method for extracting these gases from FRIB will provide access to their radio-halogen daughters which are important for nuclear medicine research, such as Positron Emission Tomography (PET) and Auger therapy.^1^

Radioactive noble gases can be accessed in FRIB’s beam-dump water through a process called “isotope harvesting”.^1^ The gases can be harvested from the ∼5000 L N_2_-filled headspace of a gas–liquid separator (GLS) tank, which is inherently saturated with water vapor at 100% relative humidity. Expected production rates at FRIB will lead to picobar partial pressures of ^76^Kr, ^77^Kr, and ^122^Xe. Therefore, any noble gas extraction process must be capable of removing trace levels of noble gases from a large-volume matrix of “wet” N_2_.

Temperature swing adsorption (TSA) is one promising approach for noble gas harvesting, where trace gases are trapped on the adsorbent at a low temperature and then released at a high temperature.^3^ The performance of a TSA process is governed by the temperature dependence of the partition of the adsorbate on the adsorbent. For trapping noble gas radioisotopes from the GLS headspace gas at FRIB, the adsorbent must also have high selectivity for noble gases over N_2_, high stability against moisture, and high stability during thermal cycling. Because the high moisture content in the GLS presents an over-icing problem for the TSA traps, the adsorption material must operate above the dew point of conventional desiccants (*T* > 233.15 K). One promising class of materials is metal–organic frameworks (MOFs), several of which exhibit noble gas adsorption at noncryogenic temperatures.^4–7^

MOFs consist of metal centers coordinated with organic linkers to form three-dimensional crystalline porous structures.^8,9^ These versatile porous materials are gaining interest for applications in radionuclide detection and separations.^10^ By varying the metal centers and organic linkers, a MOF can be tailored for adsorption of a specific noble gas.^11–13^ The mechanism for the adsorption of inert gases in MOFs is based on the van der Waals interaction, where the strongest absorption is expected when the kinetic diameter of the gas is slightly smaller than the MOF pore size. In this model, adsorption increases with increasing polarizability of the gas molecule.^14,15^ Several recent publications showed promising performance for scrubbing radioisotopes of Kr and Xe from nuclear waste gas streams utilizing various MOFs.^4,6,8,14,15^ For instance, Banerjee et al. showed that a calcium-based, microporous MOF (SBMOF-1) with 4,4-sulfonyldibenzoate linkers is by far the most promising MOF for adsorption of trace Xe and Kr from air.^8^ SBMOF-1 has rhomboidal-shaped pores with a 5.0 Å opening diameter, similar to the kinetic diameter of Kr (3.7 Å) and Xe (4.1 Å).^16,17^ An interested reader is directed to the publication of Banerjee et al.^8^ for visual representations of Xe adsorbed in a pore of SBMOF-1. In our recent paper on the scaled-up production of SBMOF-1 (up to ∼400 g in a batch), we showed high moisture stability up to 75% relative humidity during a long-term usage (∼194 days).^18^ This is due to the unique structure of SBMOF-1, which does not have an open metal coordination site, mitigating the coordination of water from the air.^19^ Meanwhile, adsorption isotherms for N_2_ on SBMOF-1 show relatively small uptake, even at relatively high N_2_ pressures.^20^ This makes SBMOF-1 a strong candidate for noble gas radioisotope harvesting from the moist N_2_ carrier gas at the FRIB.

Another adsorbent candidate used in this study is activated charcoal (AC). The AC has an extensive history of utilization for scrubbing radioactive noble gases from effluent streams.^21,22^ It is a highly porous material with a high surface area and a variety of pore dimensions, structures, and distributions.^23^ It also shows great mechanical, radiation, thermal stability, and resistance toward moisture.^24^ Additionally, it is commercially available in various engineered forms such as pellets and granules.

Designing a TSA process for isotope harvesting at FRIB requires determination of the partition coefficient for Kr and Xe on the candidate adsorbents at select temperatures in the presence of N_2_. Experimentally measured noble gas uptake can be utilized to evaluate adsorbent performance and design extraction methodology. The data can also be utilized to determine the adsorption equilibrium constant, *K­(T)*, across a range of temperatures. This allows for derivation of the enthalpy of adsorption, Δ*H*, from a fully experimental standpoint which complements the available literature values for Δ*H* for Kr-SBMOF-1, which are computationally calculated.^7,25^ Furthermore, the Van ’t Hoff equation presumes that Δ*H*, Δ*S*, and any possible activation barriers to reach equilibrium are all independent of temperature, leaving enough uncertainty to motivate experimental determination.^26^

The purpose of this work was to experimentally measure partition coefficients for trace Kr and Xe on SBMOF-1 and AC in a N_2_ carrier at varying temperatures (*T*). To achieve this, we developed a closed-loop, fixed-volume gas handling system that allowed for the continuous circulation of gas mixtures across temperature-controlled SBMOF-1 and AC adsorption beds. The equilibrium partial pressures of the gases, measured via continuous quadrupole mass spectrometry, were used to find adsorbate partition coefficients on each adsorbent. These experimentally established partition coefficients were then used to determine the enthalpy of adsorption and to predict the performance of SBMOF-1 and AC beds for the isotope harvesting application at FRIB. Furthermore, adsorption data were used to calculate *K­(T)* for Kr on SBMOF-1 and provide an experimental value for the enthalpy of adsorption.

## Methods

### Closed-Loop System (CLS) for Adsorption Studies

Gas adsorption studies were performed by utilizing a custom-made, fixed-volume, closed-loop system (CLS). The purpose of the CLS was to allow equilibration between a fixed mass of analyte gas (e.g., Kr in an N_2_ gas mixture) and a temperature-controlled adsorption bed, while measuring the equilibrium partial pressure of the analyte. The volume of the CLS is 1038 ± 33 mL, which was measured by expansion of gas into a syringe. The schematic of the CLS is shown in Figure 1.

**Figure 1:**
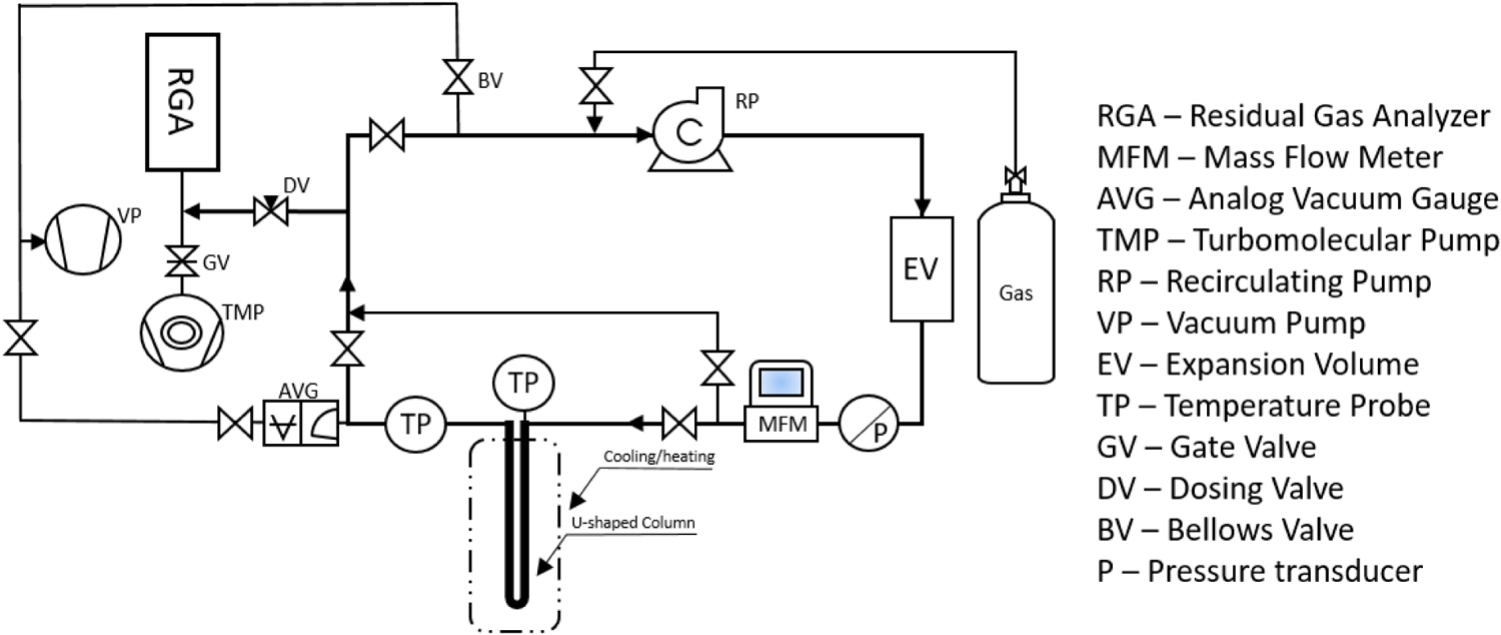
Schematic of the CLS for gas adsorption studies.

For each experiment, a known amount of adsorbent (typically 0.11–2 g) was packed into the U-shaped column (see Figure 1) and the system was hermetically sealed. The adsorbent was then regenerated by heating it to over 373.15 K while continuously pumping vacuum at <40 mTorr, for approximately 1 h. Then, the desired dry gas mixture (∼1000 ppm (ppm) Kr or Xe in N_2_) was introduced to reach total mechanical pressure of ∼1.35 bar. The gas was cycled through the system for 5 min, all while maintaining the adsorption bed temperature at ≥373.15 K. Gases were circulated in the CLS with a DIA-Vac B-series pump by Air Dimensions, Inc. with a maximum flow rate of 3.9 L·min^–1^.

Following equilibration at high temperature, the adsorption bed was cooled to ambient temperature, typically around 293.15 K, over the course of 60 min. Then, the adsorption column was submerged into a cooling bath at a select temperature and allowed to equilibrate for 36 min. The cooling bath was later removed to allow the adsorption bed to warm up back to the ambient temperature and allowed 40 min to equilibrate. The equilibration times were optimized by observing thermal equilibration kinetics in blank runs.

The partial pressures of the gases in the system were continuously monitored with a gas analysis system, based on a quadrupole mass spectrometer, which closely resembles the Noble Gas Membrane Inlet Mass Spectrometer.^27^ Gases are analyzed by a Stanford Research Systems Residual Gas Analyzer (RGA200). The high vacuum for the RGA200 is provided by a Pfeiffer HiCube turbomolecular pump. Gas from the CLS enters the high vacuum of the RGA200 via a Pfeiffer gas dosing valve (EVN 116). SAES St707 getter pellets inside the vacuum system are heated to 523.15 K to reduce the partial pressures of reactive gases (O_2_, N_2_), allowing for the quantification of noble gases at atmospheric concentrations.

Several variables were measured continuously during the following process:

- Total mechanical pressure: PX3224 model pressure transmitter (±0.35% measurement accuracy), manufactured by ifm electronic, Inc.
- Temperature of the adsorption bed: DSTPA1213212 model thermocouple by DIGI-STEM (±1.1 K tolerance) centered within the adsorbent.
- Overall temperature of the gas in the CLS: PRTXI-1/4N-1/8–6-IO thermocouple manufactured by OMEGA Engineering, Inc.

#### Materials Utilized for Adsorption Studies

SBMOF-1 was synthesized and activated following the procedure in our previous paper.^18^ The synthesized SBMOF-1 was pressed into pellets utilizing a stainless-steel pellet press die and a hydraulic press. Typically, 1 g of powdered SBMOF-1 was pressed at 1500 PSIG for 5 min and then broken into smaller pieces. The resulting mixed aggregate was sieved to obtain granules with mesh 40–20 (425–850 μm). The Norit ROW 0.8 mm pelletized AC is commercially available and was purchased from Sigma and used without any further modifications. The gas mixtures for the adsorption studies, 1016(2) ppm of Kr balance N_2_, and 999(2) ppm of Xe balance N_2_ were obtained from Advanced Specialty Gases.

#### Calculation of the Partition Coefficients and Adsorption Equilibrium

First-order adsorption equilibrium was considered between noble gas atoms, NG_(g)_, the substrate, S_(s)_, and the adsorbed state, NG-S_(s)_ via

$${\mathrm{NG}}_{\left(g\right)}+S_{\left(s\right)}⇌\mathrm{NG}‐S_{\left(s\right)}$$

The partition coefficient for trace noble gas on adsorbent can be described by the following equation

1 $$\mathrm{partition}\;\mathrm{coefficient}=\frac{n}{\mathrm{Pm}}$$

where *n* is the molar amount of noble gas adsorbed, *P* is the partial pressure of the noble gas at equilibrium in (bar), and *m* represents the mass of adsorbent used in (g).

The Langmuir-type adsorption model was used to determine the adsorption equilibrium constant, *K­(T)*, which can be defined in terms of the single components of the reversible adsorption/desorption process

2 $$K\left(T\right)=\frac{k_{a}}{k_{d}}$$

where the rate constants for the adsorption and desorption processes are represented by *k*_a_ and *k*_d_, respectively. At equilibrium, the rate of adsorption is equal to the rate of desorption; therefore, the rate equation can be set equal to solve for $\frac{k_{a}}{k_{d}}$ . Since we operate at equilibrium, we can derive equation *K­(T)* from the above-mentioned relationship to obtain

3 $$K\left(T\right)=\frac{\theta \mathrm{RT}}{P\left(1-\theta \right)}$$

Detailed derivation of eq 3 can be found in McQuarrie et al.^26^ The number of occupied adsorption sites, θ, can be defined as

4 $$\theta =\frac{n}{\mathrm{mc}}$$

where *n*_a_ is the moles of adsorbed gas, *m* is the mass of adsorbent, and *c* is the saturation capacity of the adsorbent for a given gas. The value of *c* used in this work is 1.4 mmol·g^–1^·bar^–1^.^8^ By substitution of eq 4 into eq 3, *K­(T)* becomes

5 $$K\left(T\right)=\frac{\mathrm{nRT}}{P\left(\mathrm{mc}-n\right)}$$

In this work, we used two-component gas mixtures for adsorption studies; therefore, eq 6 should be further extended to account for N_2_ adsorption. Consequently, the adsorption equilibrium constant for noble gas from bulk N_2_, *K­(T)*, becomes

6 $$K\left(T\right)=\frac{n_{a}\mathrm{RT}}{P\left(\mathrm{mc}-n_{a}-n_{b}\right)}$$

where *n*_a_ and *n*_b_ represent moles of adsorbed noble gas and N_2_, respectively. Note that for SBMOF-1, it is assumed that both noble gas and N_2_ are competing for the same adsorption sites.

The uncertainties in partition coefficient and *K­(T)* were determined by propagation of the uncertainty in each of the independent variables, then adding them in quadrature. The RGA exhibited low-frequency noise (baseline drift) with a time constant that was comparable to the length of the experiments. Therefore, the uncertainty in RGA signals was determined from the variability in measurements obtained when the adsorption bed was at room temperature. The uncertainty in the RGA signal was found to be 2.5% for Kr and 5.6% for Xe.

## Results and Discussion

### Material Characterization

Characterization results of the synthesized SBMOF-1 via Powder X-Ray Diffraction (PXRD) and Brunauer–Emmett–Teller (BET) are in agreement with previously published data.^28^ The SBMOF-1 was further modified for adsorption studies by pressing it into granules with a hydraulic press. The PXRD and BET surface area analysis of the pressed SBMOF-1 was performed to verify the structural integrity of the MOF lattice under mechanical stress. A change in the pore structure and dimensions caused by mechanical stress would result in a shift of the diffraction patterns of the MOF. The PXRD patterns of SBMOF-1 match with the reference material, which suggests that SBMOF-1 maintained its crystal structure under the mechanical stress. In addition, BET analysis confirmed that the material retained its surface area. For additional details, including PXRD diffraction patterns and BET surface analysis results, see Figure S1 and Table S1 in the Supporting Information.

In this study, we performed multiple adsorption and regeneration cycles on both SBMOF-1 and AC. To validate their stability against temperature stress and numerous adsorption and regeneration cycles, both adsorbents were characterized post-CLS adsorption studies. The SBMOF-1 was analyzed using PXRD and BET surface area analysis, which showed that MOF retained its characteristic diffraction patterns and surface area. These results suggest that the SBMOF-1 was stable over numerous adsorption and regeneration cycles and temperature stress caused by the temperature swings ranging from −195 to 373 K. The NORIT ROW charcoal was tested based on the BET surface area analysis. The change in the surface area of the AC after the noble gas adsorption studies was insignificant, which supported that the material retained its surface area after multiple adsorption and regeneration cycles as well as temperature swings. The diffraction pattern of the material post adsorption studies, as well as the surface area analysis results, are available in Supporting Information Figure S1 and Table S1.

### Determination of the Enthalpy of Adsorption for Kr on SBMOF-1 and Selectivity for Kr over N_2_

The experimentally determined adsorption equilibrium constants for Kr on SBMOF-1 are depicted in Figure 2. To determine the enthalpy of adsorption for Kr on SBMOF-1, the *K­(T)* values were fitted to the Van’t Hoff equation

7 $$K\left(T\right)=Ae^{\left(-\Delta H/\mathrm{RT}\right)}$$

**Figure 2:**
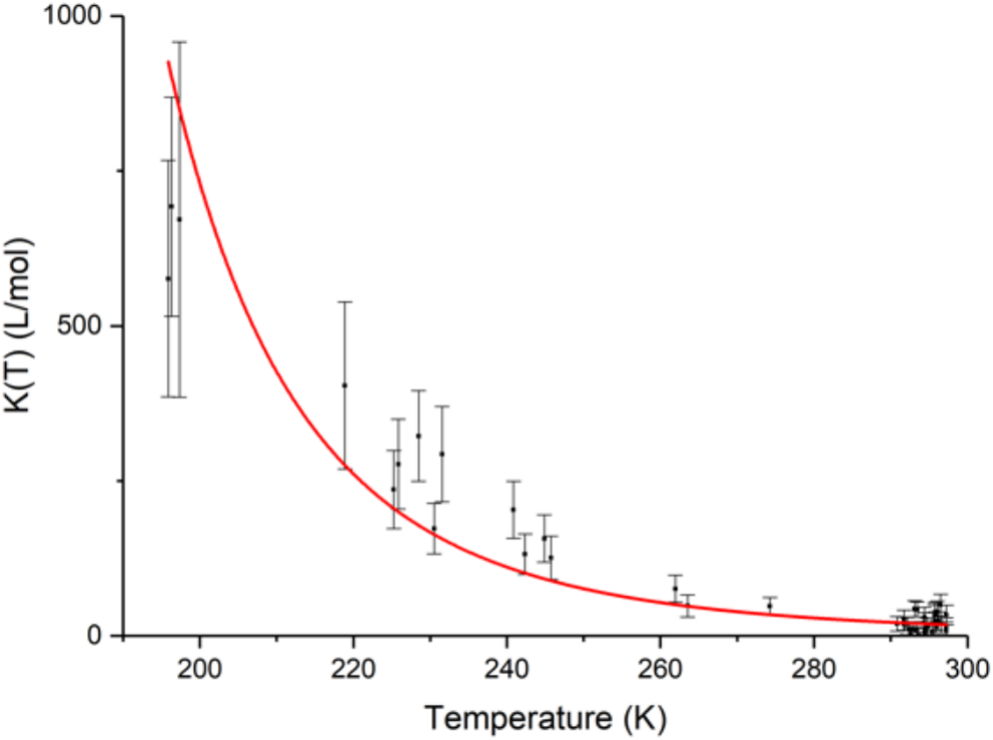
Adsorption equilibrium constants for Kr on SBMOF-1 plotted as a function of temperature. The curve is an error-weighted fit obtained by fitting data points to eq 7 following the chi-square minimization method. The red curve represents Van’t Hoff fit to the data.

where Δ*H* is the enthalpy of adsorption and *A* is a constant that depends on the entropy of adsorption, Δ*S*.^29^ In the Van’t Hoff approximation, Δ*H* and *A* (via Δ*S*) are taken to be temperature-independent and activation barriers are neglected. The Van’t Hoff fit, obtained using *A* and Δ*H* as fitting parameters in the tested temperature range of 195–293 K, is depicted in Figure 2 as a red curve. The enthalpy of adsorption approximated from the curve fit is reported in Table 1 along with the previously reported value, which was determined computationally. All of the calculated *K­(T)* values along with their uncertainties are tabulated in the Supporting Information (Table S2). Generally, we observe the expected trend of higher in magnitude adsorption equilibrium constants at lower temperatures, which agrees with the theoretical basis.^26,29,30^

**Table 1:** List of Experimentally and Computationally Determined Enthalpies of Adsorption for Kr on SBMOF-1 Compared with Two Other MOFs That Showed Promising Noble Gas Adsorption Performance^a^

MOF	Δ*H* krypton (kJ·mol^–1^)	K_H_ @ 298 K (mmol·g^–1^·bar^–1^)	Refs.
SBMOF-1	–19 ± 1^b^		this work
	–27.21^c^	2.37	Qian et al.,^13^ Banerjee et al.^8^
HKUST-1	–9.2,^b^ −19.7^c^	1.44	Farrusseng et al.,^34^ Banerjee et al.^8^
IRMOF-1	–9.8,^b^ 10.9^c^	0.51	Farrusseng et al.,^34^ Banerjee et al.^8^

a The Henry’s law constants at room temperature are also included to highlight the performance of SBMOF-1 in Kr adsorption. K_H_ was not measured in this work because these experiments were conducted in the presence of carrier N_2_.

b Experimental.

c Computational.

The fit in Figure 2 for the adsorption of Kr on SBMOF-1 across wide temperature ranges reveals some underperformance in the adsorption properties of SBMOF-1 at *T* = *∼*195 K when compared to the *K­(T)* at *T* < 220 K. There are several factors that suggest the Van’t Hoff equation may not be valid under these conditions. For example, activation barriers for gases to reach the adsorption sites become increasingly insurmountable as the temperature is lowered, and these are neglected in equilibrium calculations.^31–33^ In the case of SBMOF-1, the pore dimension is similar to the kinetic diameter of Kr; therefore, the activation barrier may be highly sensitive to temperature variations due to thermal expansion of the MOF. Thermal expansion can also alter Δ*H* because Δ*H* is dependent on the adsorption-site geometry. Similar temperature-dependent deviations were observed by Fernandez et al. when studying Xe and Kr adsorption on FMOF-Cu.^31^ Alternatively, at lower temperatures, capillary condensation can occur, which obstructs further diffusion of the gas molecules with the micropores of the SBMOF-1, effectively lowering the accessible adsorption sites.

The enthalpy of adsorption determined in this work, −19 ± 1 kJ·mol^–1^, is significantly smaller than the previously reported computationally determined value of −27.21 kJ·mol^–1^, see Table 1. The reference computational values were determined by using density functional theory (DFT). It is possible that DFT does not accurately describe weak guest–host interactions responsible for noble gas adsorption on adsorbents. Therefore, disagreements between the experimental and theoretical measures are expected. There are other possibilities for such a deviation since the adsorption studies herein were performed over a wide temperature range. Therefore, this deviation can also arise due to the same influences described above, which cause temperature dependency in the enthalpy of adsorption and kinetic barriers.

The selectivity for Kr over N_2_, *S*_NG/N_2__, was also calculated for SBMOF-1 at several temperatures using the following equation

8 $$S_{\mathrm{NG}/N_{2}}=\frac{n_{a,NG}/n_{a,N_{2}}}{X_{NG}/x_{N_{2}}}$$

where *X*_NG/XN_2__ represents the molar ratio of the noble gas to N_2_ in the gas mixture and *n*_a,NG_/*n*_a,N_2__ is the molar fraction of adsorbed noble gas to adsorbed N_2_. Experimentally determined selectivity values are listed in Table 2. In the conditions where N_2_ gas acts as a carrier and therefore is present in a bulk amount, the selectivity was determined to be around 20. Such high selectivity for Kr shows that SBMOF-1 has the potential for the selective extraction of trace levels of noble gas from the N_2_ environment.

**Table 2:** Selectivity for Kr over N_2_ Measured at Various Temperatures Utilizing 1016 ppm of Kr Balance N_2_ Gas Mixture at Approximately 1.35 Bar, Compared to Other Prospective MOFs for Noble Gas Adsorbents^a^,^b^

MOF	*S*_Kr/N_2__	Refs.
SBMOF-1	28 ± 8, 22 ± 7, 18 ± 6^b^	This Work
SIFSIX-3-Cu	24.38^c^	Elsaidi et al.^6^
HKUST-1	3.6^c^	Parker et al.^5^

a Measured at 196.3, 228.5, and 240.8 K, respectively.

b Measured at 298 K and 1 bar.

c Obtained from a single-component adsorption isotherm at 298 K and 1 bar.

### Partition Coefficients and Radioactive Noble Gas Production

The noble gas adsorption performance of SBMOF-1 and AC in a N_2_ environment can be described by considering a single-component partition coefficient of each noble gas under given conditions. This allows us to experimentally evaluate the performance of adsorbents at conditions resembling the GLS and utilize these results to approximate radioactive noble gas extraction from the N_2_ carrier at FRIB. Equation 1 was utilized to calculate partition coefficients of both Kr and Xe on SBMOF-1 and AC at various temperatures. Since the adsorption process is thermodynamically favorable, it is expected that the noble gas partition into the adsorbed phase will increase as a result of decreasing temperature. In Figure 3, a plot of Kr partition coefficients as a function of varying temperature can be observed. As expected, the Kr partition coefficients on both SBMOF-1 and AC increase in magnitude when the temperature decreases. The temperature-driven swing in the Kr uptake on adsorbents shows that both materials can be utilized for Kr extraction from N_2_ environment utilizing the TSA method. However, the magnitude of the Kr partition coefficients shows compatible performance in Kr uptake on both adsorbents, with SBMOF-1 demonstrating slightly better adsorption performance.

**Figure 3:**
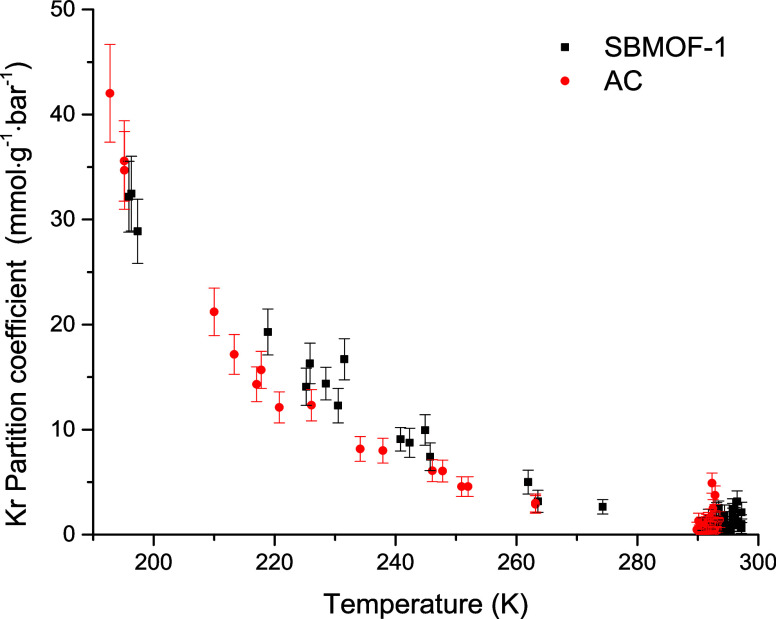
Kr partition coefficients on SBMOF-1 and AC measured in the presence of N_2_ carrier gas and plotted as a function of temperature.

Similarly, the performance of SBMOF-1 and AC was tested with Xe in N_2_ carrier gas. Due to the limitations of the CLS setup, we were not able to perform simultaneous measurements of Xe and N_2_ adsorption. The main limitation was due to the higher distribution of Xe into the adsorbent, which required us to use significantly lower amounts of the adsorbent. In combination with the much lower relative N_2_ uptake, the subtle differences in N_2_ pressure could not be observed. Therefore, we do not report on the selectivity for Xe and N_2_ as well as the *K­(T)* for Xe on SBMOF-1. However, we expect SBMOF-1 to have higher selectivity for Xe over N_2_ when compared to Kr over N_2_ for the same reasons that we observe larger partition coefficients for Xe on SBMOF-1.

From the adsorption studies, we determined Xe partition coefficients for both adsorbents. Since Xe is a more polarizable gas than Kr, due to its larger size, we observed a significantly higher distribution of the Xe gas into both adsorbents when compared to Kr. A plot of Xe uptake against temperature is given in Figure 4. In this case, SBMOF-1 shows a slightly better adsorption performance than AC, which suggests that SBMOF-1 is a more selective adsorbent material for Xe. The higher uptake of Xe by SBMOF-1 when compared to AC is due to the pore structure and dimensions that are optimized for selective Xe adsorption. In SBMOF-1, the pore diameter is similar in size to the dimensions of the guest Xe atom, creating a specific adsorption site that ensures a strong interaction of Xe with the adsorbent. On the other hand, the AC consists of various adsorption sites with pore dimensions ranging from a few nm to over 50 nm. While this makes AC a versatile adsorbent, a broad range of pore shapes and dimensions results in lower partition coefficients for Xe on AC than on SBMOF-1. The calculated Kr and Xe partition coefficients for SBMOF-1 and AC along with their uncertainties are tabulated in the Supporting Information (Tables S3–S6).

**Figure 4:**
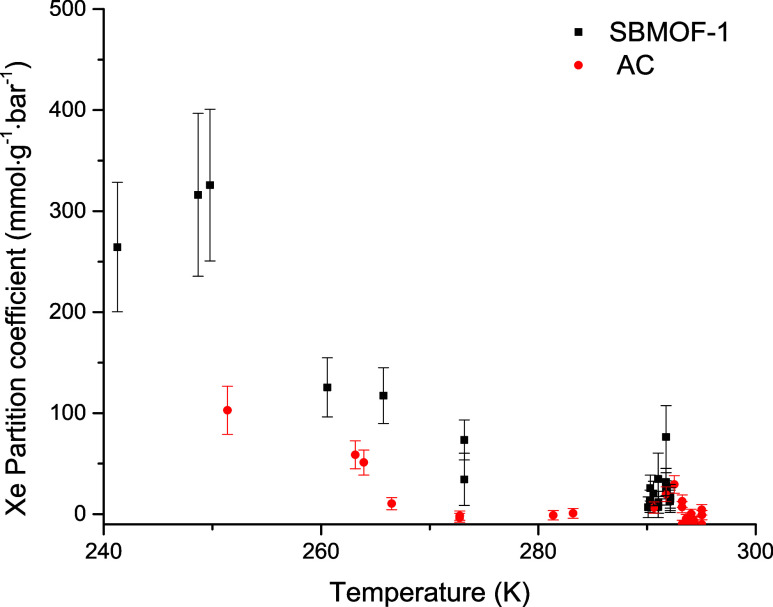
Xe partition coefficients on SBMOF-1 and AC measured in the presence of N_2_ carrier gas and plotted as a function of temperature.

The experimentally measured partition coefficients of noble gas from bulk N_2_ carrier can be used to prepare for harvesting radioactive noble gases at FRIB. During the operation of FRIB, primary beams of ^78^Kr and ^124^Xe at full power (400 kW, 200 MeV·u^–1^ or greater for nuclei with *Z* < 92) will produce GBq quantities of ^76^Kr and ^77^Kr or ^122^Xe in the water-filled beam dump (see Table 2).^1^ Because the Xe and Kr isotopes are produced from different primary beams, they will not need to be separated from each other, but only from the N_2_ carrier. As previously mentioned, these noble gas radioisotopes will be degassed in GLS. Due to the large headspace volume, the partial pressure of these radioactive gases will be in the pbar range. Either SBMOF-1 or AC can be equilibrated with the GLS gas to extract the Xe and Kr isotopes. At an adsorption bed temperature ranging from 246 to 250 K, the experimentally measured noble gas uptake data, converted to units of L·g^–1^ for convenient implementation, show that we can expect to extract MBq quantities of each desired isotope of the noble gas per gram of adsorbent (as listed in Table 3). This temperature range was selected because it is readily achievable with commercially available mechanical chillers, it is warmer than the anticipated dew point of the carrier gas (after in-line desiccation), yet it is cool enough to allow Xe and Kr trapping.

**Table 3:** Production of Radioactive Noble Gases at FRIB and Expected Extraction Performance of the SBMOF-1 and Activated Charcoal Calculated from the Experimental Partition Coefficients Measured between 240 and 250 K

		SBMOF-1		AC	
Radionuclide	Production (MBq·L^–1^)	Partition coefficient (L·g^–1^)	Extraction (MBq·g^–1^)	Partition coefficient (L·g^–1^)	Extraction (MBq·g^–1^)
^76^Kr	14.3	0.18^a^	2.6	0.15^c^	2.1
^77^Kr	93.0		16.8		13.8
^122^Xe	32.4	7.94^b^	267.2	6.12^d^	198.5

a Measured at 246 K.

b Measured at 250 K.

c Measured at 246 K.

d Measured at 247 K.

SBMOF-1 is expected to extract larger quantities of noble gases than activated charcoal per mass of adsorbent. While the difference in performance of the two adsorbents is not substantial for Kr radioisotopes, SBMOF-1 is predicted to recover ∼30% more Xe than the equivalent mass of activated charcoal. Therefore, SBMOF-1 can be viewed as a more favorable adsorbent for Xe extraction under the conditions that are expected at FRIB. It is important to note, however, that the bulk commercial availability of AC may far outweigh the benefits in adsorption for SBMOF-1. The extracted quantities of noble gas radioisotopes can be increased by scaling up the amount of adsorbent used. For reference, about 5 MBq of ^76^Br, the radioactive daughter of ^76^Kr, is needed to perform a single preclinical murine positron emission tomography (PET) scan.^35^ By performing temperature swing cycles with multiple traps, the gases can be continuously removed from the GLS headspace, allowing much greater quantities to be extracted.

## Conclusions

The performances of SBMOF-1 and activated charcoal for Kr and Xe adsorption from carrier nitrogen were evaluated across a wide range of temperatures using a custom, fixed-volume closed-loop gas adsorption system. The adsorption data were utilized to determine the adsorption equilibrium constants for Kr on SBMOF-1. The enthalpy of adsorption was experimentally determined to be −19 ± 1 kJ·mol^–1^. We observed that Kr on SBMOF-1 below 220 K shows deviations from the Van’t Hoff equation. Several possible physical processes, such as capillary condensation, activation barriers, or framework flexibility, were discussed as potential reasons for observed behaviors. Calculated partition coefficients for Kr and Xe from bulk N_2_ at varying temperatures on SBMOF-1 and AC showed that TSA can be utilized for noble gas radioisotope extraction by taking advantage of the difference in noble gas uptake at different temperatures. Valuable quantities of noble gas radioisotopes can be extracted from the effluent gas streams at FRIB using both SBMOF-1 or AC at 100 g range. For this purpose, the SBMOF-1 is ∼30% more efficient by mass than activated charcoal for harvesting xenon, but economic considerations may favor activated charcoal for noble gas radioisotope harvesting at FRIB.

## Supplementary Material



## Notes

PXRD diffraction patterns and BET analysis of the materials used in this work, all of the experimentally determined adsorption constants, and equilibrium constants (PDF)
