# Supplementary material for: Adsorption Studies of Dilute Krypton and Xenon from Nitrogen on SBMOF‑1 and Activated Charcoal for Applications in Isotope Harvesting
Source: J Phys Chem C Nanomater Interfaces. 2025 Jul 21;129(30):13768–75. doi: 10.1021/acs.jpcc.5c02812 (PMC12319903; doi:10.1021/acs.jpcc.5c02812)
Supplement: Supplementary file 1 [file jp5c02812_si_001.pdf]

SUPPORTING INFORMATION

Adsorption Studies of Dilute Krypton and Xenon from Nitrogen on SBMOF-1 and Activated Charcoal for Applications in Isotope Harvesting

Vladyslav S. Bodnar,[a,b] Chloe R. Kleinfeldt,[a,b] Sung Ho Kim,[c] Noelle R. Catarineu,[c] Chirag K. Vyas,[b] Ate Visser,[c] Gregory W. Severin[a,b,*]

[a]Department of Chemistry, Michigan State University, East Lansing, Michigan 48824, United States
[b]Facility for Rare Isotope Beams, Michigan State University, East Lansing, Michigan 48824, United States
[c]Lawrence Livermore National Laboratory, Livermore, California 94550, United States
*Corresponding author severin@frib.msu.edu

Material analysis

After the adsorption studies, the SBMOF-1 and AC were characterized to validate their stability using powder X-ray diffraction (PXRD) analysis and Brunauer-Emmett-Teller (BET) analysis. MOFs were analyzed with PXRD to validate that the material retained its structural integrity at varying temperatures using a Bruker D8 Davinci diffractometer. The surface area of both adsorbents were evaluated by BET analysis, using a Micromeritics ASAP 2020 instrument, to determine whether each retained its surface area.

The PXRD analysis of the pressed SBMOF-1 granules suggest that they maintained their crystal structure under the mechanical stress exerted on it by the hydraulic press. Additionally, the analysis of the post-adsorption experiments material showed that the SBMOF-1 is stable over the course of many adsorption cycles at conditions described in this work (see **Figure S1**).

The BET surface area analysis revealed that the surface area of the pressed MOF is slightly lower than the previously reported for powdered SBMOF-1. The surface area of the pressed MOF did not decrease following several adsorption cycles (see **Table S1**). Both the PXRD and BET analysis of the SBMOF-1 suggest that the material is stable towards mechanical stress and multiple adsorption/desorption cycles. The activated charcoal was characterized only via BET surface area analysis. The surface area of NORIT ROW activated charcoal was measured to be over 10 times higher than that of the SBMOF-1. The change in the surface area of the activated charcoal after the noble gas adsorption studies was insignificant, suggesting stability of the material throughout multiple adsorption and desorption cycles.

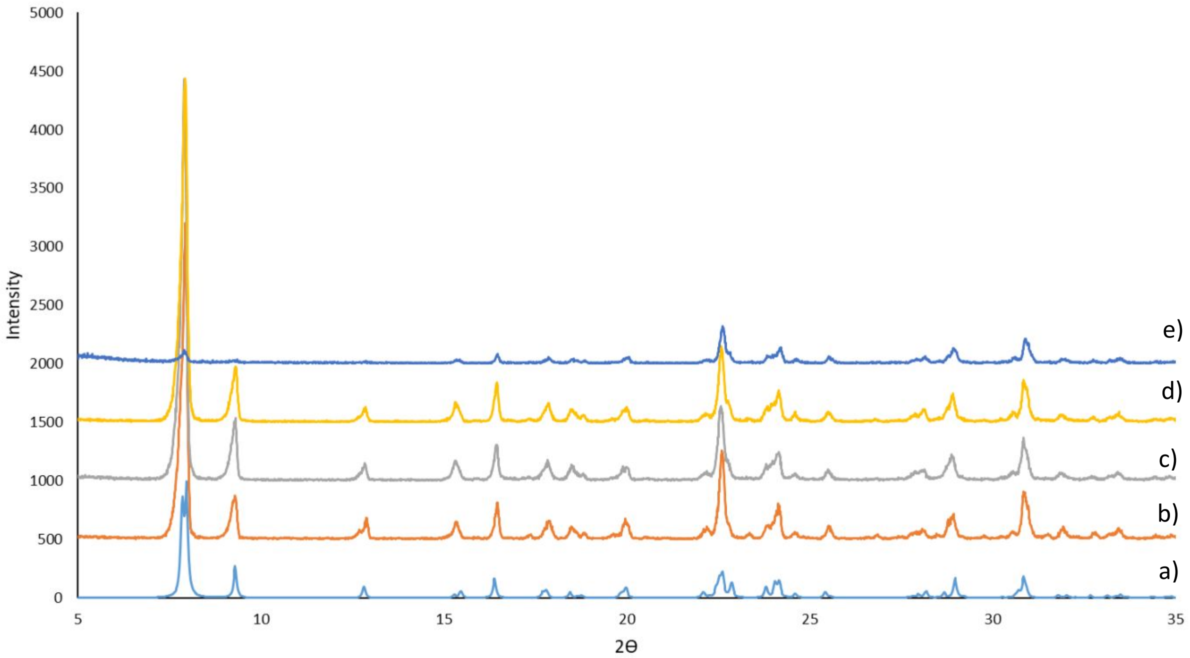

Figure S1. Powder XRD diffraction patterns of a) SBMOF-1 generated from crystal structure published in ccdc,[1] b) SBMOF-1 as synthesized, c) pressed SBMOF-1, d) pressed SBMOF-1 post Kr adsorption studies, e) pressed SBMOF-1 post Xe adsorption studies.

Table S1. Surface area of the adsorbents before and after adsorption studies, measured using BET method.

Material	Surface area ($m^2 \cdot g^{-1}$)
Pressed SBMOF-1	100 ± 6
Pressed SBMOF-1 post Kr adsorption	126 ± 8
Pressed SBMOF-1 post Xe adsorption	115 ± 6
NORIT ROW activated charcoal	1311 ± 72
NORIT ROW activated charcoal post Kr adsorption	1131 ± 64
NORIT ROW activated charcoal post Xe adsorption	1158 ± 62

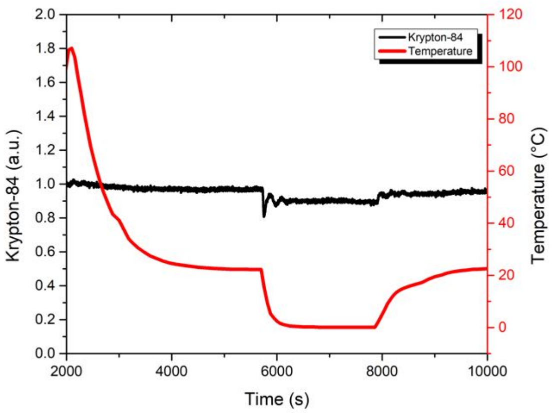

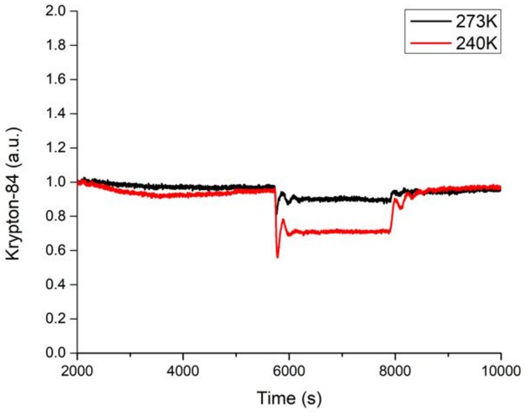

Figure S2. Adsorption curves for Kr on SBMOF-1. The left plot represents a baseline subtracted and normalized Kr partial pressure in the system with the adsorption bed temperature overlayed. The plot on the right represents a baseline subtracted and normalized Kr partial pressure in the system measured at two different temperatures.

Table S2. List of all experimentally measured adsorption constants *K(T)* for Kr on SBMOF-1.

Temperature (K)	K(T) ($L \cdot mol^{-1}$)	Uncertainty
195.9	576	191
196.3	692	177
197.4	672	286
218.9	404	135
225.2	236	63
225.9	277	72
228.5	322	73
230.5	173	41
230.5	173	41
231.5	293	77
240.8	203	46
242.3	132	33
244.9	157	38
245.7	126	35
261.9	76	22
263.5	48	18
274.3	48	14
290.8	19	12
291.5	0	13
291.7	19	11
291.7	25	16
292.1	19	11
292.2	-1	13
292.5	0	14
292.5	9	14
292.6	1	12
292.6	1	12
292.9	10	10
293.1	43	13
293.3	8	15
293.5	43	12
293.7	0	12
293.7	0	12
294.3	21	16
294.4	30	16
294.4	5	13

294.4	11	10
294.8	14	15
295.4	6	13
295.7	34	17
295.7	38	16
295.8	24	15
295.9	15	10
296.1	39	17
296.2	24	14
296.5	49	18
296.5	18	14
296.9	-9	12
297.2	15	14
297.2	9	13
297.2	34	16

Table S3. List of all experimentally measured partition coefficients for Kr on SBMOF-1.

Temperature (K)	Partition Coefficient ($mmol \cdot g^{-1} \cdot bar^{-1}$)	Uncertainty
195.9	32.2	3.4
196.3	32.5	3.6
197.4	28.9	3.1
218.9	19.3	2.2
225.2	14.1	1.8
225.9	16.3	1.9
228.5	14.4	1.6
230.5	12.3	1.6
231.5	16.7	2.0
240.8	9.1	1.1
242.3	8.7	1.4
244.9	10.0	1.5
245.7	7.4	1.3
261.9	5.0	1.1
263.5	3.2	1.0
274.3	2.6	0.7
290.8	1.0	0.6
291.5	0.0	0.9
291.7	1.0	0.6
291.7	1.5	0.9
292.1	1.1	0.6
292.2	-0.1	0.9
292.5	0.0	0.9
292.5	0.6	0.9
292.6	0.1	0.9
292.9	0.5	0.6
293.1	2.4	0.7
293.3	0.5	0.9
293.5	2.5	0.7
293.7	0.0	0.9
294.3	1.3	0.9
294.4	1.9	1.0
294.4	0.3	0.9
294.4	0.6	0.6
294.8	0.8	0.9
295.4	0.4	0.9

295.7	2.0	1.0
295.7	2.4	1.0
295.8	1.6	0.9
295.9	0.9	0.6
296.1	2.4	1.0
296.2	1.6	0.9
296.5	3.2	1.0
296.5	1.3	0.9
296.9	-0.6	0.8
297.2	1.0	0.9
297.2	0.6	0.9
297.2	2.1	1.0

Table S4. List of all experimentally measured partition coefficients for Kr on AC.

Temperature (K)	Partition Coefficient ($mmol \cdot g^{-1} \cdot bar^{-1}$)	Uncertainty
192.8	42.0	4.6
195.1	35.6	3.8
195.2	34.7	3.7
210.0	21.2	2.3
213.3	17.2	1.9
217.0	14.3	1.7
217.8	15.7	1.8
220.8	12.1	1.5
226.1	12.3	1.5
234.2	8.2	1.2
237.9	8.0	1.2
246.1	6.1	1.0
247.8	6.1	1.0
250.9	4.6	0.9
252.0	4.6	0.9
263.1	2.9	0.8
263.2	3.0	0.9
289.8	0.5	0.7
290.1	1.3	0.8
290.2	0.2	0.7
290.6	0.9	0.7
290.7	0.8	0.7
291.4	0.1	0.7
291.4	1.0	0.7
291.4	1.2	0.7
291.7	1.0	0.7
291.7	0.1	0.7
291.7	0.8	0.7
291.7	0.3	0.7
291.7	1.4	0.8
291.7	1.1	0.7
292.1	1.7	0.8
292.1	0.5	0.7
292.1	0.9	0.7
292.1	0.5	0.7
292.1	0.1	0.7

292.1	0.9	0.7
292.1	1.2	0.7
292.2	0.8	0.7
292.3	4.9	0.9
292.4	0.7	0.7
292.5	2.5	0.8
292.5	0.8	0.7
292.8	1.5	0.8
292.8	1.4	0.7
292.8	3.8	0.9
292.9	0.6	0.7
293.0	0.1	0.7
293.2	0.5	0.7
293.3	0.9	0.7
293.6	1.4	0.8

Table S5. List of all experimentally measured partition coefficients for Xe on SBMOF-1.

Temperature (K)	Partition Coefficient (mmol·g^{-1}·bar^{-1})	Uncertainty
196.3	885	247
233.2	510	161
241.2	264	64
248.7	316	81
249.8	326	75
260.6	125	29
265.7	117	28
273.2	34	26
273.2	73	20
289.3	-37	18
290.1	7	10
290.3	13	11
290.3	26	13
290.6	20	12
291.0	35	26
291.0	7	10
291.0	-55	16
291.1	12	11
291.4	-20	19
291.7	-14	20
291.7	32	13
291.8	76	31
291.8	28	13
291.8	21	12
292.1	13	11
292.1	18	12
292.1	15	11

Table S6. List of all experimentally measured partition coefficients for Xe on AC.

Temperature (K)	Partition Coefficient (mmol·g^{-1}·bar^{-1})	Uncertainty
251.4	102.8	23.8
263.2	58.7	13.8
263.9	51.2	12.4
266.5	10.4	6.0
272.8	-3.7	4.4
272.8	-1.5	4.6
281.4	-1.1	4.6
281.4	-1.1	4.6
283.2	0.8	4.8
290.6	6.7	5.6
290.7	6.4	5.5
291.8	19.8	7.3
292.5	29.5	8.7
293.2	7.0	5.5
293.2	12.8	6.3
293.2	-9.8	3.7
293.6	-11.1	3.6
293.6	-4.5	4.2
294.0	-3.3	4.4
294.1	0.3	4.8
294.2	-6.0	4.1
294.2	-6.0	4.1
294.3	-6.9	4.0
295.0	4.4	5.2
295.1	-0.9	4.6
295.1	-0.9	4.6
295.1	-10.1	3.6

References

1. Plonka, A. M.; Chen, X.; Wang, H.; Krishna, R.; Dong, X.; Banerjee, D.; Woerner, W. R.; Han, Y.; Li, J.; Parise, J. B. Light hydrocarbon adsorption mechanisms in two Calcium-Based microporous metal organic frameworks. *Chemistry of Materials* **2016**, *28* (6), 1636–1646. https://doi.org/10.1021/acs.chemmater.5b03792.
